# Reactive Species Interactome Alterations in Oocyte Donation Pregnancies in the Absence and Presence of Pre-Eclampsia

**DOI:** 10.3390/ijms20051150

**Published:** 2019-03-06

**Authors:** Manon Bos, Mirthe H. Schoots, Bernadette O. Fernandez, Monika Mikus-Lelinska, Laurie C. Lau, Michael Eikmans, Harry van Goor, Sanne J. Gordijn, Andreas Pasch, Martin Feelisch, Marie-Louise P. van der Hoorn

**Affiliations:** 1 Department of Pathology, Leiden University Medical Center, 2333ZA Leiden, The Netherlands; 2 Department of Obstetrics and Gynaecology, Leiden University Medical Center, 2333ZA Leiden, The Netherlands; m.l.p.van_der_hoorn@lumc.nl; 3 Department of Pathology and Medical Biology, University Medical Center Groningen, University of Groningen, 9713 GZ Groningen, The Netherlands; m.h.schoots@umcg.nl (M.H.S.); h.van.goor@umcg.nl (H.v.G.); 4 Clinical & Experimental Sciences, Faculty of Medicine, University of Southampton and Southampton General Hospital, Southampton SO17 1BJ, UK; b.fernandez@soton.ac.uk (B.O.F.); m.mikus-lelinska@soton.ac.uk (M.M.-L.); l.c.lau@soton.ac.uk (L.C.L.); m.feelisch@soton.ac.uk (M.F.); 5 Department of Immunohematology and Blood Transfusion, Leiden University Medical Center, 2333ZA Leiden, The Netherlands; m.eikmans@lumc.nl; 6 Department of Obstetrics, University Medical Center Groningen, University of Groningen, 9713 GZ Groningen, The Netherlands; s.j.gordijn@umcg.nl; 7 Department of Biomedical Research, University of Bern, 3012 Bern, Switzerland; andreas.pasch@insel.ch

**Keywords:** oocyte donation, reactive species interactome, thiols, nitric oxide, hydrogen sulfide, pre-eclampsia

## Abstract

In pregnancy, maternal physiology is subject to considerable adaptations, including alterations in cardiovascular and metabolic function as well as development of immunological tolerance towards the fetus. In an oocyte donation pregnancy, the fetus is fully allogeneic towards the mother, since it carries both oocyte donor antigens and paternal antigens. Therefore, oocyte donation pregnancies result in an immunologically challenging pregnancy, which is reflected by a higher-than-normal risk to develop pre-eclampsia. Based on the allogeneic conditions in oocyte donation pregnancies, we hypothesized that this situation may translate into alterations in concentration of stable readouts of constituents of the reactive species interactome (RSI) compared to normal pregnancies, especially serum free thiols, nitric oxide (NO) and hydrogen sulfide (H_2_S) related metabolites. Indeed, total free thiol levels and nitrite (NO_2_^−^) concentrations were significantly lower whereas protein-bound NO and sulfate (SO_4_^2−^) concentrations were significantly higher in both oocyte donation and naturally conceived pregnancies complicated by pre-eclampsia. The increased concentrations of nitrite observed in uncomplicated oocyte donation pregnancies suggest that endothelial NO production is compensatorily enhanced to lower vascular tone. More research is warranted on the role of the RSI and bioenergetic status in uncomplicated oocyte donation pregnancies and oocyte donation pregnancies complicated by pre-eclampsia.

## 1. Introduction

In pregnancy, maternal physiology is subject to considerable adaptation, including alterations in cardiovascular and metabolic function as well as development of immunological tolerance towards the fetus [1,2,3,4]. In a naturally conceived pregnancy, the fetus can be considered semi-allogeneic since it carries both paternal and maternal antigens [5]. Although the semi-allogeneic conceptus is recognized by the maternal immune system, as reflected by the presence of allo-antibodies directed against paternal antigens in the maternal circulation, the conceptus is tolerated rather than rejected [6,7,8,9]. Furthermore, fetal cells are in direct contact with maternal cells at the fetal–maternal interface [7,10,11]. 

Oocyte donation is an artificial reproductive technique that enables women without ovarian activity or diminished ovarian reserve to conceive [12]. Furthermore, women with a serious genetic disease can choose to use oocytes of a healthy donor. In an oocyte donation pregnancy, the fetus is fully allogeneic towards the mother, since it carries both oocyte donor antigens and paternal antigens; this antigenic dissimilarity in unmatched oocyte donation pregnancies is comparable to the antigenic dissimilarity in unmatched organ transplantation [5]. Due to the enhanced allogeneic nature of oocyte donation, these pregnancies presumably need more or different maternal immune adaptations, compared to naturally conceived pregnancies [5,13,14,15]. The increased adaptive load on the immune system between naturally conceived and oocyte donation pregnancies may explain why the latter are more often accompanied by immune-disturbance related obstetrical complications, such as pregnancy induced hypertension and pre-eclampsia [13,15,16]. Oocyte donation pregnancies more often coincide with other risk factors for pregnancy complications, such as the self-evident need for artificial reproductive techniques, advanced maternal age, primiparity, cause of infertility, and multiple gestations [17,18,19,20,21]. However, several studies have shown that the allogeneic nature of oocyte donation itself is an independent risk factor for pre-eclampsia [16,22,23,24,25,26]. 

Pre-eclampsia is a syndrome of pregnancy that is characterised by hypertension and problems in multiple organ systems [27,28]. It is caused by a hypoxic placenta which results in an aberrant placental production of pro-inflammatory cytokines and anti-angiogenic factors. This translates into an enhanced systemic inflammatory status, systemic angiogenic imbalance and subsequently, in generalised endothelial dysfunction eventually resulting in the manifestation of clinical symptoms of pre-eclampsia [17,28]. Since hypoxia, inflammation and vascular stress are accompanied by an aberrant production of reactive oxygen species (ROS), as is the case in many other disease processes [29], an enhanced presence of ROS may play a central role in pre-eclampsia. The placental tissue itself is an important site of production of ROS and other reactive species; indeed, the pre-eclamptic placenta is characterised by an increased production of reactive species [30,31,32]. This aberrant placental production of ROS and reactive nitrogen species (RNS) may conceivably contribute to systemic endothelial dysfunction in pre-eclampsia [33,34,35,36] and is likely to be accompanied by corresponding changes in the production of reactive sulfur species (RSS).

The gasotransmitters nitric oxide (NO), carbon monoxide (CO) and hydrogen sulfide (H_2_S) are important for vascular adaptations in pregnancy [37]. Gasotransmitters are known to regulate angiogenesis and vascular tone and their production is linked to the regulation of antioxidant status; the abnormal production of these small molecules is associated with pre-eclampsia [34,38,39,40]. Many of these reactive small molecules (some of which belong to the group of gasotransmitters) are known to chemically interact with each other [41]. Furthermore, ROS, RNS and RSS also react with other biological targets such as thiol (SH) groups of enzymes, transcription factors and ion channels, which enables sensing and adaptation processes of cells and tissues to changes in environmental conditions and/or metabolic demand. This interaction of reactive species with other small molecules and biological targets has been defined as the “reactive species interactome” (RSI) [41].

In allograft organ transplantation, systemic redox status predicts graft survival and mortality [42,43,44], and reduced oxidative damage is associated with a better kidney transplant outcome [45]. Based on the allogeneic conditions in oocyte donation pregnancies, we hypothesized that the RSI status is affected in oocyte donation pregnancies. Therefore, we evaluated the RSI status, measured by quantifying circulating total free thiols, total 8-iso-prostaglandin F_2a_, and metabolites of the NO and H_2_S pathway in naturally conceived and oocyte donation pregnancies, in the absence and presence of concomitant pre-eclampsia.

## 2. Results

### 2.1. Study Participants, Pregnancy Characteristics and Fetal Characteristics

Seventy-nine pregnant women were included in this case-control study; 23 women experienced an uncomplicated naturally conceived pregnancy, 24 women had a naturally conceived pregnancy complicated by pre-eclampsia, 27 women experienced an uncomplicated oocyte donation pregnancy, and 5 women had an oocyte donation pregnancy complicated by pre-eclampsia. Characteristics of study participants are summarized in Table 1.

Maternal age was significantly lower in uncomplicated naturally conceived pregnancies and naturally conceived pregnancies complicated by pre-eclampsia compared to uncomplicated oocyte donation pregnancies and oocyte donation pregnancies complicated by pre-eclampsia. BMI, smoking habits, gravidity, and parity were comparable among groups. Moreover, obstetrical history was comparable among groups. Only the number of previous miscarriages was significantly different across groups (overall test, *p* < 0.05). Women experiencing an uncomplicated pregnancy were more likely to have had uncomplicated pregnancies before. Of the uncomplicated pregnancies, three women developed gestational hypertension after a naturally conceived pregnancy and four women who were pregnant after oocyte donation had hypertension. None of these women fulfilled the International Society for the study of Hypertension in Pregnancy (ISSHP) criteria for the diagnosis of pre-eclampsia; the women with gestational hypertension had no other obstetric problems, and their fetal growth was adequate for the gestational age [28]. Diastolic blood pressure in uncomplicated oocyte donation pregnancies was significantly higher compared to naturally conceived uncomplicated pregnancies. None of the women in our study had gestational diabetes or a history of gestational diabetes. Fetal sex was comparable among groups. Birthweight of fetuses born after a pregnancy complicated by pre-eclampsia was significantly lower compared to uncomplicated pregnancies. 

### 2.2. Presence of Oxidative Stress

**Figure 1:**
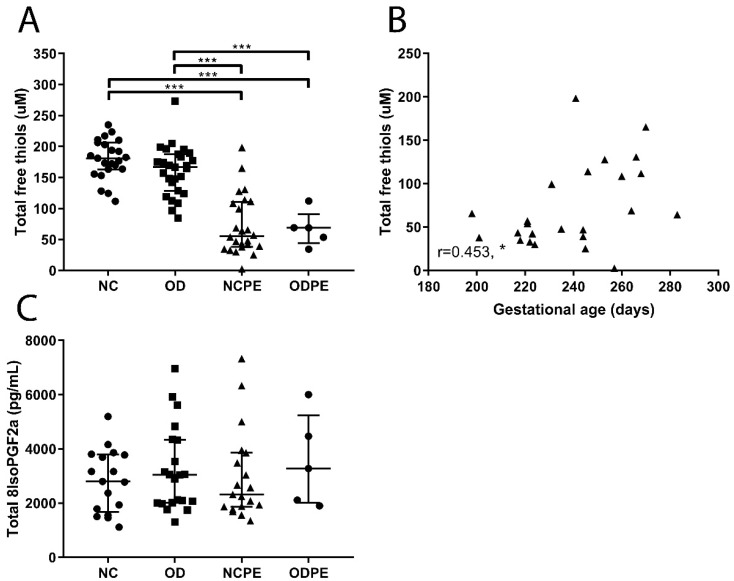
Indicators of oxidative stress. (**A**) Total free thiol concentrations were lower in pregnancies complicated by pre-eclampsia compared to controls and uncomplicated oocyte donation pregnancies (all, *p* < 0.001). (**B**) Total free thiol concentrations were positively correlated with gestational age in naturally conceived pregnancies complicated by pre-eclampsia (r = 0.453, * *p* < 0.05). (**C**) Total 8-iso-prostaglandin F_2a_ concentrations in serum did not differ between groups. NC, uncomplicated naturally conceived pregnancies; OD, uncomplicated oocyte donation pregnancies; NCPE, naturally conceived pregnancies complicated by pre-eclampsia; ODPE, oocyte donation pregnancies complicated by pre-eclampsia. * *p* < 0.05, *** *p* < 0.001.

The concentrations of both total free thiols and total 8-iso-prostaglandin F_2a_ in blood are a measure of redox stress. Protein-bound free thiols may function as a buffer system for a number of reactive species and may also fulfil specific transport functions. Total (free and bound) 8-iso-prostaglandin F_2a_ is a product of lipid oxidation and indicative of systemic oxidative stress. Total free thiol concentrations were lower in pregnancies complicated by pre-eclampsia, both after naturally conceived pregnancies and oocyte donation pregnancies, compared to uncomplicated pregnancies of either type (Figure 1A, *p* < 0.001). Furthermore, total free thiol concentrations revealed a weak positive association with gestational age in naturally conceived pregnancies complicated by pre-eclampsia (Figure 1B; *r* = 0.453, *p* < 0.05). Serum levels of total 8-iso-prostaglandin F_2a_ did not differ among groups (Figure 1C).

### 2.3. Nitric Oxide Pathway

**Figure 2:**
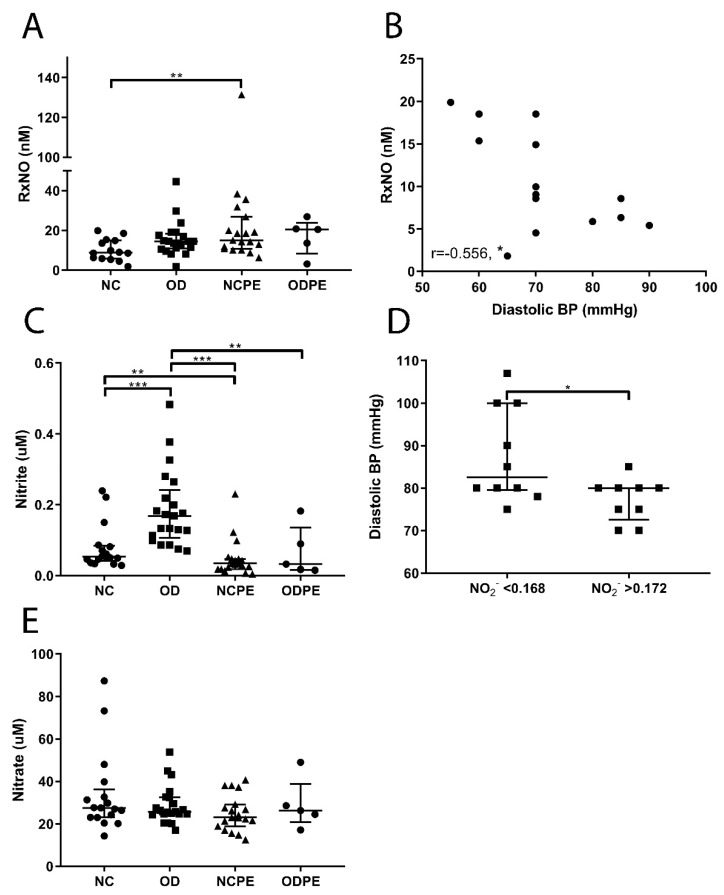
Readouts of the nitric oxide (NO) pathway. (**A**) The concentrations of protein bound NO (RxNO) were higher in pregnancies complicated by pre-eclampsia (** *p* < 0.01). (**B**) Diastolic blood pressure was inversely associated with RxNO in uncomplicated naturally conceived pregnancies (r = −0.556, * *p* < 0.05). (**C**) Nitrite (NO_2_^−^) concentrations were lower in naturally conceived pregnancies complicated by pre-eclampsia and oocyte donation pregnancies complicated by pre-eclampsia compared to uncomplicated naturally conceived pregnancies and uncomplicated oocyte donation pregnancies, respectively (** *p* < 0.01 and *** *p* < 0.001). Nitrite levels were higher in uncomplicated oocyte donation pregnancies compared to uncomplicated naturally conceived pregnancies (*** *p* < 0.001). (**D**) Patients in the uncomplicated oocyte donation group were divided into two groups based on the nitrite serum levels: One group contained serum nitrite values below the median of 0.168 uM, while the other group contained serum nitrite values above the median. Diastolic blood pressure was found to be higher in women with serum nitrite values below the median compared to women with a serum nitrite values above the median (* *p* < 0.05). (**E**) Nitrate (NO_3_^−^) concentrations did not differ between groups. NC, uncomplicated naturally conceived pregnancies; OD, uncomplicated oocyte donation pregnancies; NCPE, naturally conceived pregnancies complicated by pre-eclampsia; ODPE, oocyte donation pregnancies complicated by pre-eclampsia. * *p* < 0.05, ** *p* < 0.01, *** *p* < 0.001.

Nitric oxide (NO) is an important cellular messenger produced by the oxidation of L-arginine by nitric oxide synthases (NOS). In addition to its acute local production, NO bound to circulating proteins, such as serum albumin, can function as a storage and transport system of bioactive NO [46,47]; nitrite (NO_2_^−^) and nitrate (NO_3_^−^) are the major oxidation products of NO, but are also contained in food. The concentration of protein bound NO (RxNO) was higher in pregnancies complicated by pre-eclampsia (Figure 2A, *p* < 0.01). Interestingly, diastolic blood pressure correlated inversely with RxNO concentrations in women with an uncomplicated naturally conceived pregnancy (Figure 2B). Nitrite concentrations were lower in naturally conceived pregnancies complicated by pre-eclampsia and oocyte donation pregnancies complicated by pre-eclampsia compared to uncomplicated naturally conceived pregnancies and uncomplicated oocyte donation pregnancies, respectively. Also, circulating nitrite was higher in uncomplicated oocyte donation pregnancies compared to uncomplicated naturally conceived pregnancies (Figure 2C). Moreover, diastolic blood pressures were higher in women with an uncomplicated oocyte donation pregnancy and serum nitrite levels below the median of 0.168 µM compared to women with a serum nitrite level above the median (Figure 2D, *p* < 0.05). Nitrate concentrations were not significantly different across groups (Figure 2E).

### 2.4. Hydrogen Sulfide Pathway

**Figure 3:**
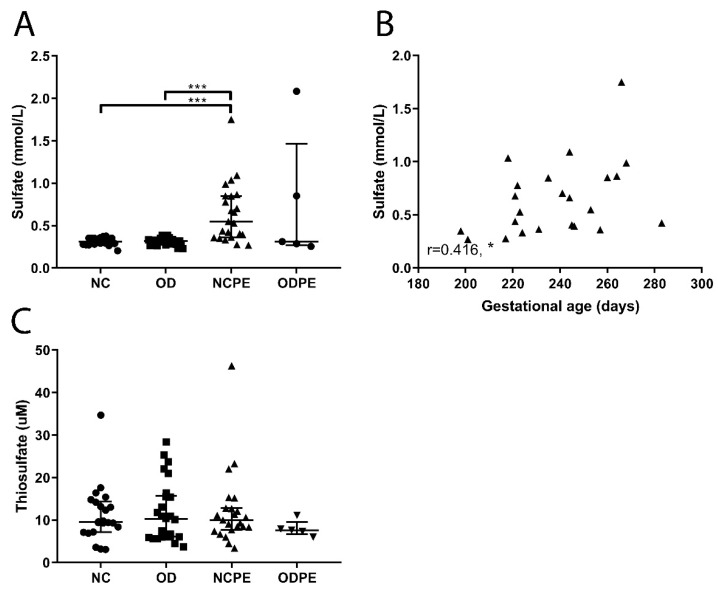
Readouts of the hydrogen sulfide (H_2_S) pathway. (**A**) Sulfate (SO_4_^2−^) values were higher in naturally conceived pregnancies complicated by pre-eclampsia compared to uncomplicated naturally conceived pregnancies and uncomplicated oocyte donation pregnancies (*** *p* < 0.001). (**B**) Sulfate (SO_4_^−^) values correlated positively with gestational age in naturally conceived pregnancies complicated by pre-eclampsia (r = 0.416, * *p* < 0.05). (**C**) Thiosulfate (S_2_O_3_^2−^) values did not differ between groups. NC, uncomplicated naturally conceived pregnancies; OD, uncomplicated oocyte donation pregnancies; NCPE, naturally conceived pregnancies complicated by pre-eclampsia; ODPE, oocyte donation pregnancies complicated by pre-eclampsia. * *p* < 0.05, *** *p* < 0.001.

Thiosulfate (S_2_O_3_^2−^) and sulfate (SO_4_^2−^) are oxidative metabolites of hydrogen sulfide (H_2_S) and dietary constituents. Serum sulfate concentrations were higher in both naturally conceived pregnancies and oocyte donation pregnancies complicated by pre-eclampsia compared to uncomplicated naturally conceived pregnancies and uncomplicated oocyte donation pregnancies (Figure 3A; both, *p* < 0.001). Sulfate (SO_4_^−^) concentrations correlated positively with gestational age in naturally conceived pregnancies complicated by pre-eclampsia (Figure 3B; r = 0.416, *p* < 0.05). By contrast, thiosulfate (S_2_O_3_^2−^) concentrations did not differ across groups (Figure 3C).

## 3. Discussion

In this observational study, we evaluated the RSI status in naturally conceived and oocyte donation pregnancies in the absence and presence of pre-eclampsia. Naturally conceived and oocyte donation pregnancies complicated by pre-eclampsia appear to be characterized by systemic redox stress; total free thiol levels and nitrite concentrations were significantly lower, whereas protein bound NO and sulfate concentrations were significantly higher in pregnancies complicated by pre-eclampsia compared to uncomplicated pregnancies. Some of these changes have been described before in women with pre-eclampsia [48,49,50]. However, the literature is inconsistent [51,52]. The hypoxic placenta is known to produce higher fluxes of ROS [30,31,32] and the same is true for the endothelium of women with pre-eclampsia [36]. Since superoxide anions (O_2_^−^), an important ROS, directly react with NO to produce peroxynitrite (ONOO^−^), enhanced oxidative stress typically translates into lower NO bioavailability. Consistent with this paradigm, a reduced bioavailability of NO is known to be involved in the development of generalised endothelial dysfunction and hypertension in women with pre-eclampsia [35]. More recently, oxidative stress-induced S-glutathionylation of endothelial nitric oxide synthase has been suggested to account for impaired NO production in the placenta [53]. We have shown that the concentrations of free thiols and sulfate appear to positively correlate with gestational age in naturally conceived pregnancies complicated by pre-eclampsia. While these associations are modest, they would seem to be consistent with the notion that perturbations in systemic redox status are tightly linked to the development of pre-eclampsia; a shorter gestational age in women with pre-eclampsia is mainly due to iatrogenic birth (caesarean section or induction of labour) based on the fetal or maternal condition, and is therefore always directly associated with the severity of the disease. Both source and utilization of sulfate deserve further study in this context, as this anion is not only the final oxidation product of H_2_S, but also an important nutrient for human growth and development [54]. This is particularly important in view of the limited capacity of fetal tissues to produce sulfate, thereby relying almost entirely on its supply via the maternal circulation.

In oocyte donation pregnancies, the fetus could be completely allogeneic to the gestational carrier [5]. This condition is thus comparable with the situation of antigenic dissimilarity present in organ transplantation [5,13]. Based on the immunogenic dissimilarity in oocyte donation, and what is known from the redox status in transplantation, one might expect more oxidative stress in oocyte donation pregnancies. However, total free thiol and total 8-iso-prostaglandin F_2a_ concentrations were not changed in uncomplicated oocyte donation pregnancies compared to uncomplicated naturally conceived pregnancies. Curiously, increased nitrite concentrations were observed in uncomplicated oocyte donation pregnancies compared to naturally conceived pregnancies. Circulating nitrite levels are a reliable biomarker of endothelial NO production [55,56]. NO is a potent vasodilator and an important regulator of vascular tone, and upregulated NO production could reduce blood pressure and the development of hypertension [37,43,57]. However, this would be expected to be accompanied by a concomitant increase in circulating nitrate (NO_3_^−^) concentrations. Diastolic blood pressure was slightly higher in uncomplicated oocyte donation pregnancies compared to uncomplicated naturally conceived pregnancies (Table 1, *p* < 0.05). Upregulation of NO/nitrite in oocyte donation could be a compensatory mechanism to limit further increases in blood pressure in these women. This is supported by the inverse association between diastolic blood pressure and protein bound NO (RxNO) concentrations in uncomplicated naturally conceived pregnancies. RxNO functions as a NO storage pool [47], and lower RxNO levels may reflect higher utilization of this alternative source of NO. Furthermore, diastolic blood pressure is higher in women with an uncomplicated oocyte donation pregnancy with nitrite serum concentrations below the median compared to women with concentrations above the median (Figure 2D). The importance of NO in the regulation of blood pressure in pregnancy is shown in an animal experiment where NO production was blocked in virgin and pregnant rats. The blockage of NO synthases resulted in the development of hypertension in pregnant rats as well as in virgin rats. Moreover, pregnant animals are more sensitive for NO synthesis blockade than non-pregnant animals [58]. Thus, higher serum nitrite concentrations in women with uncomplicated oocyte donation pregnancies could indicate a role of this NO metabolite in the regulation of vascular tone, which might function as a compensatory mechanism in uncomplicated oocyte donation pregnancies to prevent a further increase in blood pressure caused by the genetic incompatibility between mother and fetus in oocyte donation pregnancies. However, we cannot exclude other possible explanations, such as the increased maternal age in women pregnant after oocyte donation. 

Study limitations include the small group size of women with oocyte donation complicated by pre-eclampsia, which was a direct consequence of the definition of case selection criteria. We were concerned that the results could be influenced by the duration of sample storage and therefore opted to select samples from a defined study period to avoid this potential influence. Another limitation of our study is that maternal age and gestational age were different between study groups. Maternal age is an important risk factor for pre-eclampsia and could influence the rate of production and metabolism of reactive species [27,41,59]. Since women pregnant after oocyte donation are usually older, it is difficult to match maternal age between naturally conceived and oocyte donation pregnancies. Nevertheless, none of the measured molecules was associated with maternal age. Likewise, due to the presence of pre-eclampsia (and the consequent preterm delivery), matching for gestational age is difficult. A preterm control group does not exist due to the underlying factors leading to preterm delivery. Lastly, the dietary intake of nitrite, nitrate and sulfate by the study participants, which could affect the RSI status [41,60] and constitute an analytical confounding factor, was not determined.

## 4. Materials and Methods 

### 4.1. Study Participants and Biospecimen Collection

A retrospective case-control study was performed in women pregnant after oocyte donation or natural conception, and delivered in the Leiden University Medical Center (LUMC) and peripheral hospitals in the Leiden region between 2012 and 2016. Maternal age, gestational age, highest diastolic blood pressure, and presence of pre-eclampsia were documented. Different groups were clinically matched for BMI, smoking habits, gravidity, parity, and mode of delivery. This study was carried out following the rules of the Declaration of Helsinki and collection of samples was approved by the ethics committee of the Leiden University Medical Center (P13.084, 17 June 2013). All subjects gave their informed consent for inclusion before they participated in the study.

### 4.2. Clinical Definitions

Miscarriage was defined as the spontaneous loss of pregnancy within the first 24 weeks of gestation [61,62]. Termination of pregnancy was defined as a termination on a medical fetal or maternal indication within the first 24 weeks of pregnancy resulting in fetal demise (Dutch guideline on termination of pregnancy until 24 weeks of gestational age; [63]). Abortion was when pregnancy loss was induced within the first 24 weeks of gestation for social reasons [64]. IUFD was defined as fetal loss after 24 weeks of gestation. Gestational hypertension was defined as de novo hypertension (systolic blood pressure ≥ 140mmHg and/or diastolic blood pressure ≥ 90 mmHg) after gestational week 20. Pre-eclampsia was defined as de novo hypertension after gestational week 20 and new onset of proteinuria (≥300 mg/24 h or a spot urine protein creatinine ratio ≥ 30 mg/mmol), renal insufficiency, liver disease, neurological problems, haematological disturbances or fetal growth restriction [65]. A term pregnancy was defined as childbirth after 37 weeks of pregnancy, and preterm birth was defined as birth between 24–37 weeks of gestation. Small for gestational age was defined as a birth weight below the 10th percentile for gestational age according to the Dutch reference curves for birth weight by gestational age [66]. Gestational diabetes is defined as onset or first recognition of abnormal glucose tolerance during pregnancy.

### 4.3. Measurements

Serum samples were collected in the third trimester and stored at −80 °C until measurements. The concentrations of the following analytes of the RSI were measured in serum: Total free thiols, protein bound NO (RxNO), nitrite (NO_2_^−^), nitrate (NO_3_^−^), 8-iso-prostaglandin F_2a_ (isoprostanes), sulfate (SO_4_^2−^) and thiosulfate (S_2_O_3_^2−^).

#### 4.3.1. Colorimetric Detection of Total Free Thiol Groups

Free thiols were detected using Ellman’s reagent as described previously [67,68]. Briefly, serum samples were diluted 1:3 with Tris buffer (0.1 M, pH 8.2), and background absorbance was read at 412 nm using a microplate reader with reference at 630 nm. Then, 10 µL 5,5’-Dithio-bis(2-nitrobenzoic acid) (DTNB; 3.8mM, Ellman’s Reagent) diluted in phosphate buffer (0.1 M, pH 7) was added. The samples were incubated with DTNB for 20 min at room temperature, sample absorbance was measured again at 412 nm with 630 nm reference and compared to a calibration curve constructed for L-cysteine under identical conditions. 

#### 4.3.2. Assessment of Nitroso Species via Gas Phase Chemiluminescence Detection

Protein bound nitric oxide (RxNO) concentrations in the serum were quantified using reductive denitrosation by iodine-iodide with subsequent detection of liberated NO by gas-phase chemiluminescence, as described elsewhere [46]. Samples were thawed for 30 min at room temperature (RT) in the presence of N-ethylmaleimide (NEM; 10 mM final concentration), and nitrite was removed by reaction with acifified sulphanilamide before injection into the septum-sealed reaction chamber containing triiodide in glacial acetic acid as detailed previously [46].

#### 4.3.3. Determination of Nitrite and Nitrate 

The HPLC method used employs ion chromatography with on-line reduction of nitrate to nitrite and subsequent post-column derivatization with the Griess reagent (ENO-20, Eicom, Kyoto, Japan) [46]. Samples were subjected to deproteinization using ice-cold methanol (1:1 *v*/*v*) followed by centrifugation. A total volume of 20 µL of sample was loaded onto the column, resulting in a detection limit of 10 nM for either anion.

#### 4.3.4. Total Free 8-Iso-Prostaglandin F_2a_ Determination

Free 8-iso-prostaglandin F_2a_ was measured by competitive ELISA using a commercial assay kit (Cayman Chemical, Ann Arbor, MI, USA). The concentration of total (free + bound) 8-isoprostanes was determined after subjecting serum to alkaline hydrolysis and a final dilution of 20-fold with assay buffer before measurement.

#### 4.3.5. Sulfate Determination

Serum sulfate concentrations were quantified by means of ion-exchange chromatography after protein precipitation with methanol and a subsequent 10-fold dilution with ultrapure water. 

#### 4.3.6. Thiosulfate Determination

Thiosulfate was determined by HPLC following monobromobimane (MBB) derivatization, as previously described [69,70,71]. Briefly, 25 μL of urine was derivatized with 5 μL of 46 mM MBB, 25 μL of acetonitrile, and 25 μL of 160 mM HEPES/16 mM EDTA pH 8 buffer for 30 min in the dark. The derivatization reaction was stopped by addition of 50 μL of 65 mM methanosulfonic acid and proteins were removed by centrifugation [71].

### 4.4. Statistical Analysis

Normally distributed continuous data were analysed using ANOVA and the least significant differences as a post-hoc test. A log transformation of the data was performed based on the Levene’s test and/or the distribution of the residuals. When a log transformation did not improve our analysis the Tamhane correction for unequal variances was used. Categorical data were analysed using a Pearson chi-square test, and when applicable a Fisher’s exact test for subgroup analysis. For correlations between RSI metabolites clinical outcomes, Spearman’s rho was used. A *p*-value below 0.05 was considered statistically significant. All analyses were performed using the SPSS statistics software (Version 23, IBM Nederland B.V., Amsterdam, The Netherlands).

## 5. Conclusions

In conclusion, alterations in the RSI status are present in naturally conceived pregnancies as well as in oocyte donation pregnancies complicated by pre-eclampsia, and the concentrations of both total free thiols and sulfate are associated with the severity of pre-eclampsia in naturally conceived pregnancies. Furthermore, changes in the RSI status are also seen in uncomplicated oocyte donation pregnancies compared to uncomplicated naturally conceived pregnancies. In this study, a possible compensatory mechanism via the NO-pathway was found in uncomplicated oocyte donation pregnancies. This exploratory study is the first that elucidated possible changes in select readouts of the RSI in oocyte donation pregnancies. More research is warranted on the role of the RSI in uncomplicated oocyte donation pregnancies and oocyte donation pregnancies complicated by pre-eclampsia. Given that mitochondrial function and cellular energetics are intimately interconnected to the extracellular redox status and modulated by several RSI constituents at multiple levels [41], it is possible that the latter plays an important role in fetal development and the prevention of complications in pregnancy.

## Figures and Tables

**Table 1:** Patient characteristics, obstetrical history, pregnancy characteristics and fetal characteristics.

ART	Naturally Conceived	Oocyte Donation	Naturally Conceived	Oocyte Donation	
Complication	Uncomplicated	Uncomplicated	Pre-Eclampsia	Pre-Eclampsia	
**Maternal characteristics**	*n* = 23	*n* = 27	*n* = 24	*n* = 5	**Overall sig.**
Maternal Age (years), mean (SD)	31.8 (5.2)	41.1 (6.4) *	30.8 (5.8)	40.0 (7.2) #	*p* < 0.001 ^a^
BMI (kg/m^2^), median (range)	25.1 (18.8–37.8)	24.3 (16.9–31.6)	24.6 (19.3–36.8)	25.3 (20.7–27.2)	ns ^a^
Smoking, number (%)	2 (9.5)	1 (5)	6 (26.1)	1 (20.0)	ns ^c^
Gravidity, median (range)	1 (1–8)	2 (1–8)	1 (1–5)	1 (1–10)	ns ^a^
Gravidity 1, number (%)	12 (52.2)	9 (36.0)	14 (58.3)	3 (60.0)	ns ^c^
Parity, median (range)	0 (0–3)	0 (0–1)	0 (0–4)	0 (0–0)	ns ^a^
Parity 0, number (%)	13 (56.5)	15 (60.0)	19 (79.2)	5 (100)	ns ^c^
**Obstetrical history**	*n* = 11	*n* = 15	*n* = 10	*n* = 2	
Previous pregnancies	*n* = 26	*n* = 39	*n* = 15	*n* = 10	
Miscarriage, number (%)	8 (30.8)	27 (69.2)	7 (46.7)	8 (80.0)	*p* < 0.05 ^c^
Abortion, number (%)	1 (3.8)	0	1 (6.7)	0	ns ^c^
EUG, number (%)	0	3 (7.7)	0	2 (20)	ns ^c^
TOP, number (%)	0	0	1 (6.7)	0	ns ^c^
Pre-term birth, number (%)	0	2 (5.1)	1 (6.7)	0	ns ^c^
IUFD, number (%)	0	0	1 (6.7)	0	ns ^c^
Gestational hypertension, number (%)	0	2 (5.1)	1 (6.7)	0	ns ^c^
Pre-eclampsia, number (%)	0	0	1 (6.7)	0	ns ^c^
Gestational diabetes, number (%)	0	0	0	0	
Pregnancies without complications, number (%)	17 (65.4)	5 (12.8) *	3 (20) *	0	*p* < 0.05 ^c^
**Pregnancy characteristics**					
ART, number (%)	0	27 (100) *	0	5 (100) ^#^	*p* < 0.001 ^c^
Hypertension, number (%)	3 (13.6)	4 (17.4)	24 (100) *	5 (100) ^&^	*p* < 0.001 ^c^
Highest diastolic BP, (mmHg), mean (SD)	74 (10)	82 (10) *	101 (9) *	101 (11) ^&^	*p* < 0.001 ^a^
Proteinuria, number (%)	0	0	23 (95.8) *	5 (100) ^&^	*p* < 0.001 ^c^
Pre-eclampsia, number (%)	0	0	24 (100) *	5 (100) ^&^	*p* < 0.001 ^c^
HELLP-syndrome, number (%)	0	0	4 (16.7) *	0	*p* = 0.022 ^c^
Gestational age (days), median (range (days))	275 (269–290)	279 (231–290)	243 (198–283) *	217 (204–270) ^&^	*p* < 0.001 ^a^
Preterm birth, number (%)	0	1 (4.2)	18 (75.0) *	4 (80.0) ^&^	*p* < 0.001 ^c^
Gestational diabetes, number (%)	0	0	0	0	
Delivery, vaginal, number (%)	6 (26.1)	11 (45.8)	12 (50.0)	1 (25.0)	
Delivery, CS, number (%)	17 (73.9)	13 (54.2)	12 (50.0)	3 (75.0)	ns ^c^
Twin, number (%)	0	2 (7.4)	0	1 (20.0)	ns ^c^
**Fetal characteristics**	*n* = 23	*n* = 29	*n* = 24	*n* = 6	
Sex, male/female (%male)	16/7 (69.6)	10/18 (35.7) *	14/10 (58.3)	3/3 (50.0)	ns ^c^
Birthweight (gram), median (range)	3455 (2445–4415)	3500 (1611–4500)	2372 (705–4030) *	1319 (1100–3855) ^&^	*p* < 0.001 ^a^
Small for gestational age, number (%)	1 (4.3)	5 (19.2)	10 (41.7) *	3 (50.0)	*p* < 0.01 ^c^

ART, artificial reproductive technique; BP, blood pressure; CS, caesarean section; EUG, extra uterine gravidity; IUFD, intra uterine fetal demise; ns, not significant; TOP, termination of pregnancy. * significantly different compared to uncomplicated naturally conceived pregnancies, *p* < 0.05. ^#^ significantly different compared to naturally conceived pregnancies complicated with pre-eclampsia, *p* < 0.05. ^&^ significantly different compared to uncomplicated oocyte donation pregnancies, *p* < 0.05. Statistical tests: ^a^ ANOVA, Post-Hoc test, LSD or Tamhane when applicable, ^c^ Pearson chi-square, when applicable Fisher’s exact test for subgroup analysis.

## Abbreviations

H_2_S	Hydrogen sulfide
NO	Nitric oxide
NO_2_^−^	Nitrite
NO_3_^−^	Nitrate
ROS	Reactive oxygen species
RNS	Reactive nitrogen species
RSS	Reactive sulfur species
RSI	Reactive species interactome
RxNO	Protein bound nitric oxide
S_2_O_3_^2−^	Thiosulfate
SO_4_^2−^	Sulfate
